# Use of The International Classification of Functioning, Disability and Health (ICF) as a conceptual framework and common language for disability statistics and health information systems

**DOI:** 10.1186/1471-2458-11-S4-S3

**Published:** 2011-05-31

**Authors:** Nenad Kostanjsek

**Affiliations:** 1 World Health Organization, Classifications, Terminology and Standards, Geneva, Switzerland

## Abstract

A common framework for describing functional status information is needed in order to make this information comparable and of value. The World Health Organization’s International Classification of Functioning, Disability and Health (ICF), which has been approved by all its member states, provides this common language and framework. The article provides an overview of ICF taxonomy, introduces the conceptual model which underpins ICF and elaborates on how ICF is used at population and clinical level. Furthermore, the article presents key features of the ICF tooling environment and outlines current and future developments of the classification.

## Introduction

The approval of the International Classification of Functioning, Disability and Health (ICF) [1] by the World Health Assembly in May 2001 has marked a paradigm shift in the way health and disability are understood and measured. Until recently, “health” has only been seen as the opposite of death or disease. Traditional health indicators have mostly focused on mortality and morbidity. On the other hand, “disability” has been seen as an unrelated entity, either as a medical issue of bodily impairments such as blindness and deafness or as an imposed restriction on the individual that hinders him/her from taking part in daily life activities. ICF has brought these concepts into a comprehensive whole of multiple dimensions of human functioning synthesizing biological, psychological, social and environmental aspects. ICF, thus, presents health and disability in a single spectrum. Traditionally these areas have been thought separately and at times put into polarization. However, a detailed analysis of the domains that make up health and disability shows that these two basic constructs are in fact different manifestations of the same domains of functioning such as seeing, hearing and many others.

Formulating human functioning in multiple dimensions under a biopsychosocial view is not new to a number of medical fields, such as rehabilitative medicine, mental health, physical, occupational and speech and language therapy, and in nursing home and home care settings. What is new is that with the adoption of ICF we have a globally agreed-on conceptual framework and common language to document and code functional status information.

## ICF taxonomy

As a classification, ICF systematically groups different domains for a person in a given health condition (e.g. what a person with a disease or disorder does do or can do) in two parts. Part 1 deals with Functioning and Disability, while Part 2 covers Contextual Factors. Each part has two components:

1. Components of Functioning and Disability

The Body component comprises two classifications, one for the functions of the body systems, and one for the body structures. The chapters in both classifications are organized according to the body systems.

The Activities and Participation component covers the complete range of domains denoting aspects of functioning from both an individual and a societal perspective.

2. Components of Contextual Factors

A list of Environmental Factors is the first component of Contextual Factors. Environmental factors have an impact on all components of functioning and disability and are organized in sequence from the individual’s most immediate environment to the general environment.

Personal Factors is also a component of Contextual Factors but they are not classified in ICF because of the large social and cultural variance associated with them.

The components of Functioning and Disability in Part 1 of ICF can be expressed in two ways. On the one hand, they can be used to indicate problems (e.g. impairment, activity limitation or participation restriction summarized under the umbrella term disability), on the other hand they can indicate non-problematic (i.e. neutral) aspects of health and health-related states summarized under the umbrella term functioning.

These components of functioning and disability are interpreted by means of four separate but related constructs. These constructs are operationalized by using qualifiers. Body functions and structures can be interpreted by means of changes in physiological systems or in anatomical structures. For the Activities and Participation component, two constructs are available: capacity and performance.

Table 1 gives an overview of the ICF components with their definitional characteristics and classification at chapter level.

**Table 1:** ICF Taxonomy

Body Functions and Structures	
**Taxonomy of Body Functions:** (ICF Chapter) 1. Mental Functions 2. Sensory Functions and Pain 3. Voice and Speech Functions 4. Functions of the Cardiovascular, Haematological, Immunological and Respiratory Systems 5. Functions of the Digestive, Metabolic, Endocrine Systems 6. Genitourinary and Reproductive Functions 7. Neuromusculoskeletal and Movement-Related Functions 8. Functions af the Skin and Related Structures	**Taxonomy of Body Structures** (ICF Chapters) 1. Structure of the Nervous System 2. The Eye, Ear and Related Structures 3. Structures Involved in Voice and Speech 4. Structure of the Cardiovascular, Immunological and Respiratory Systems 5. Structures Related to the Digestive, Metabolic and Endocrine Systems 6. Structure Related to Genitourinary and Reproductive Systems 7. Structure Related to Movement 8. Skin and Related Structures

**Activities and Participation**	

1. Learning and Applying Knowledge 2. General Tasks and Demands 3. Communication 4. Mobility 5. Self Care 6. Domestic Life 7. Interpersonal Interactions and Relationships 8. Major Life Areas 9. Commmunity, Social and Civic Life	

**Environmental Factors**	

1. Products and Technology 2. Natural Environment and Human-Made Changes to Environment 3. Support and Relationships 4. Attitudes 5. Services, Systems and Policies	

## ICF conceptual model

As a "second generation" classification, ICF is concept driven. Apart from classifying the universe of disability, ICF also provides a conceptual framework for understanding disability.

At the core of the ICF concept of health and disability is the notion that disability is a multidimensional and universal phenomena placed on a continuum with health. Human functioning is understood as a continuum of health states and every human being exhibits one or another degree of functioning in each domain, at the body, person and society levels.

ICF conceptualises disability not solely as a problem that resides in the individual, but as a health experience that occurs in a context. Disability and functioning are, according to the ICF model, outcomes of interactions between health conditions (diseases, disorders and injuries) and contextual factors. The bio-psychosocial model embedded in the ICF broadens the perspective of disability and allows medical, individual, social, and environmental influences on functioning and disability to be examined.

Moreover, ICF is grounded in the principle of universality, namely that functioning and disability are applicable to all people, irrespective of health condition, and in particular that disability – or decrement in functioning at one or more levels – is not the mark of a specific minority class of people, but is a feature of the human condition, which is, epidemiologically speaking, over the life-span, a universal phenomena. In addition, ICF is committed to the principle of parity, which states that the functional status is not determined by background etiology, and in particular by whether one has a ‘physical’ rather than ’mental’ health condition.

**Figure 1:**
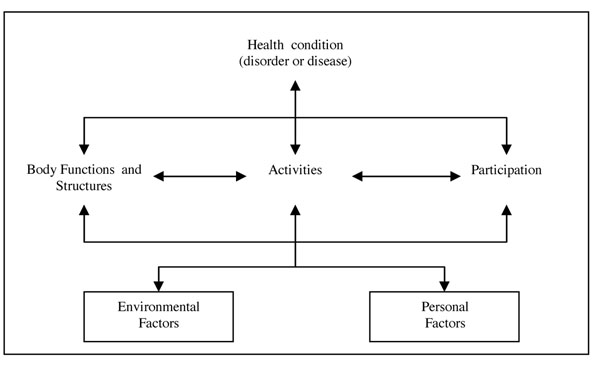
Interactions between the components of ICF

Figure 1 depicts the components of the ICF in an interactive model. The health condition (a disease or disorder) may impact functioning at 3 mutually interacting levels: in relation to the body, at the level of activities, and at the level of participation in society. The way health condition impacts functioning should also be considered within the context of environmental and personal factors.

The ICF model and its underlying principles represent a significant development from its predecessor, the International Classification of Impairment, Disability and Handicap (ICIDH) [2]. In the ICIDH, disability was understood as a limitation in the person’s activity that resulted from impairment. Neither disabilities nor handicaps could be assessed in terms of degree of severity. Environmental factors were acknowledged but not classified and no linkages between disability and health status measurement were made. Due to these limitations, ICIDH was generally viewed as flawed and so was ignored by disability data users in general and by advocates of the social model of disability (especially organizations representing persons with disabilities) in particular. In response to these and other criticisms, the ICF was developed over a seven-year period in an international collaborative process and validated by means of field trails in over 70 countries before officially being endorsed by all WHO Member States in 2001.

## ICF applications and development of a classification infrastructure

ICF was conceived as a common language and data standard, capable of being used for multiple purposes and in different settings. Since its adoption by the World Health Assembly in 2001, the ICF has been implemented in a variety of ways at various levels. The listing of examples below provides an overview of where and how the classification is used.

Applications at population level:

• Health and disability data collection in surveys of general and specific populations: ICF provides the conceptual framework and item pool for Multi-Country Studies (e.g. the Global Study on Ageing - SAGE [3]), the World Mental Health Survey (WMHS) [4], the World Health Survey (WHS) [5], the WHO Multi-Country Survey Study (MCSS) [6] as well as national surveys (e.g. Ireland [7],Chile [8], Mexico [9]).

• Data compilation and analysis: ICF-based disability prevalence and multi-domain functioning levels at global and regional level are presented in the upcoming WHO World Report on Disability and Rehabilitation. At regional level, projects like "Measurement of Health and Disability in Europe (MHADIE)" [10] applied the ICF in their analysis of population health and disability data. At country level, the ICF is used to facilitate the harmonization and comparability of data sets (e.g. Australian Health Data Dictionary) [11].

• Development of disability survey modules & question sets: the ICF framework and taxonomy is used to derive disability question sets for international and regional projects including the WHO health and disability survey module, the EUROSTAT Survey Module on “Disability and Social Integration" [12], and the Washington Group on Disability Statistics [13].

• Policy development and monitoring: international treaties and initiatives like the Millennium Development Goal (MDGs) or the UN Convention on the Rights of Persons with Disabilities (CRPD) [14] as well as national health and social policies are setting targets and indicators. For guiding policy development and monitoring, its implementation need to be matched with data sources. Pilot projects are currently exploring the use of the ICF framework and coding system for monitoring policy implementation by linking policy targets and indicators with the respective data sources [15,16].

Applications at health and social service level:

• National legislation: the ICF model and definition of disability is used as the reference standard for health- and disability-related legislation. In Germany, for example, the 9^th^ Basic Social Law (SGB IX) [17] uses ICF as reference for regulating the entitlements and service provision for people with chronic disease and disability.

• Service provision: the ICF is used in documenting and coding the functional status information for purpose of assessing patient needs, planning health and social care and for measuring the changes brought by interventions across a multitude of dimensions from body functions to personal activities, societal participation and environmental factors. The uptake of the ICF and ICF-based instruments has been particularly strong in the area of medical, social and occupational rehabilitation [18-20]. More recently we have also seen the ICF and ICF-based instruments like the WHO Disability Assessment Schedule (WHODAS 2.0) [21] being used to measure health needs and outcomes of interventions outcome across various disease areas and health care settings [22-24].

• Disability certification: various countries have or are in the process of implementing projects where ICF is used in assessing the disability status of individuals in order to determine their eligibility for health, social, or educational services [25,26]. The move to an ICF approach is driven by a broad consensus that disability should be understood as the result of a complex interaction between a person and his or her environment and not as a characteristic of an individual. Consequently, it could be presumed that eligibility should also take into account the functioning of the persons in their environment and move beyond using only one-dimensional, deficit-oriented diagnostic or body impairment labels.

To support the implementation of ICF, a wide range of application tools and training materials have been developed over the past years. For standardized cross-cultural measurement of health status, WHO has developed a new version of the Disability Assessment Schedule (WHODAS 2.0) [27] as a general measure of functioning and disability that reflects major life domains as classified in the ICF (see Table 2). Another instrument is the ICF Checklist [28,29], which is a practical translation of the ICF for clinical practice. Items from the classification were chosen by experts to list the most commonly used domains and later field tested to verify the selection and make additions of missing items. The ICF Checklist gives a thumbnail sketch of the main functioning of any individual in terms of body functions and structures, activities and participation, and environmental factors. The ICF checklist also includes diagnostic information, which enables the user to study the relationship between a health condition and the associated functioning problems. Both instruments were explicitly designed to be generic assessment tools usable in a wide range of applications aiming at data comparability across conditions and interventions. This feature constitutes the primary strength and virtue of these two instruments.

**Table 2:** The WHO Disability Assessment Schedule (WHODAS 2.0)

WHODAS 2.0 is a practical, generic assessment instrument that can measure health and disability at population level or in clinical practice [21]. WHODAS 2.0 captures the level of functioning in six domains of life: • Domain 1: Cognition – understanding and communicating • Domain 2: Mobility – moving and getting around • Domain 3: Self-care – attending to one’s hygiene, dressing, eating and staying alone • Domain 4: Getting along – interacting with other people • Domain 5: Life activities – domestic responsibilities, leisure, work and school • Domain 6: Participation – joining in community activities, participating in society.
The six domains were selected after a careful review of existing research and survey instruments, and a cross-cultural applicability study. For all six domains, WHODAS 2.0 provides a profile and a summary measure of functioning and disability that is reliable and applicable across cultures, in all adult populations. WHODAS 2.0 provides a common metric of the impact of any health condition in terms of functioning. Being a generic measure, the instrument does not target a specific disease – it can thus be used to compare disability due to different diseases. WHODAS 2.0 also makes it possible to design and monitor the impact of health and health-related interventions. The instrument has proven useful for assessing health and disability levels in the general population and in specific groups (e.g. people with a range of different mental and physical conditions). Furthermore, WHODAS 2.0 makes it easier to design health and health-related interventions, and to monitor their impact. WHODAS 2.0 is grounded in the conceptual framework of the ICF. All domains were developed from a comprehensive set of ICF items and map directly onto the ICF “Activities and Participation” component. As in the ICF, WHODAS 2.0 places health and disability on a continuum, with disability defined as “a decrement in each functioning domain”. In addition, WHODAS 2.0, like the ICF, is etiologically neutral; that is, it is independent of the background disease or previous health conditions. This feature makes it possible to focus directly on functioning and disability, and allows the assessment of functioning separately from the disease conditions. There are several different versions of WHODAS 2.0, which differ in length and intended mode of administration. The full version has 36 questions and the short version 12 questions; these questions relate to functioning difficulties experienced by the respondent in the six domains of life during the previous 30 days. The different versions can be administered by a lay interviewer, by the person themselves or by a proxy (i.e. family, friend or carer). The 12-item version explains 81% of the variance of the more detailed 36-item version. For both versions, general population norms are available.

Using ICF in specialty settings may require to focus on a particular range and granularity of ICF codes. For example, a clinician dealing with patients with depression will need a wider range of categories to identify the area of mental functions and interpersonal interactions and relationships. A speech and language therapist, on the other hand, will require detailed description of voice and speech functions and related structures.

In response to this information need in specialized clinical settings, ICF core sets [30] have been developed. Using the ICF Checklist as a starting point, the ICF Core Sets identify the most relevant ICF domains for a series of health conditions. For clinicians who choose to continue to use their existing clinical measures and map them to the ICF framework, semantic maps have been developed alongside with mapping rules and procedures.

Sensitization and training of stakeholders from health, social service and educational institutions proved to be a critical element in promoting the ICF implementation in these sectors. Over the last years, a number of ICF training tools and awareness raising and education activities on ICF were conducted [31,32]. Currently a web-based ICF e-learning tool is being developed and field tested. The introduction module of the tool is expected to be launched by the end of 2010.

## ICF developments

As a classification the ICF is meant to be a "living document" and therefore has to be kept up-to-date and developed further.

To update the ICF, WHO has established a web-based ICF update platform, which is open to any person in the general public. The platform also provides a workbench for experts of the WHO Functioning and Disability Reference Group (FDRG) and the Update and Revision Committee (URC) to process update proposals. The application collects update proposals in a structured and organized manner. This is done by asking the user to fill a form in which the user explains the proposal as well as the rationale behind the proposal. In addition to this, he/she can provide links to web pages such as publication references from PubMed or can upload documents that are relevant to the proposal. Subsequently, each proposal is reviewed online by experts from the FDRG in terms of its compliance with established criteria like: validity, reliability, added value as classification entity; consistency with ICF structure, concept and taxonomic principles; rationale and evidence base. Following the review, experts in the URC decide whether the proposal should be implemented or rejected.

An important milestone in the further development of the ICF was the publication of the ICF Children and Youth version (ICF-CY) [33] in 2007. As the first ICF-derived classification, the ICF-CY is expanding the coverage of the main ICF volume by providing specific content and additional detail to more fully cover the body functions and structures, activities and participation, and environments of particular relevance to infants, toddlers, children and adolescents. An important novelty introduced by the ICF-CY is that it captures and operationalizes the notion of the “developing child” and takes into account the following: (a) the child needs to be viewed in the context of the family; (b) the nature and forms of participation change dramatically from dependent relationships in infancy to complex life situations in adolescence; (c) mirroring developmental changes in participation, the nature and number of environments change as well; and (d) lags in emergence of functions or acquisition of skills may reflect developmental delay rather than impairments or stable limitations.

One of the most important prospects for the future development and implementation of the ICF emerge from aligning ICF and International Classification of Diseases (ICD) [34] in the context of the ongoing ICD revision process.

## Conclusions

ICF puts the notions of ‘health’ and ‘disability’ in a new light. It acknowledges that every human being can experience a decrement in health and thereby experience some disability. This is not something that happens to only a minority of humanity. ICF thus ‘mainstreams’ the experience of disability and recognizes it as a universal human experience. By shifting the focus from cause to impact it places all health conditions on an equal footing allowing them to be compared using a common metric – the ruler of health and disability.

Since its publication in 2001, the ICF has been gradually implemented in a variety of settings and sectors. At clinical level, its uptake has been most noticeable in the area of medical, social and occupational rehabilitation. The ICF information on functioning and disability enriches the diagnostic information in the ICD, providing a broader, more meaningful picture of the patient’s health, which can be used for better management decisions.

## Competing interest

The authors declare that they have no competing interests.
